# The use of Electronic Health Records to Support Population Health: A Systematic Review of the Literature

**DOI:** 10.1007/s10916-018-1075-6

**Published:** 2018-09-29

**Authors:** Clemens Scott Kruse, Anna Stein, Heather Thomas, Harmander Kaur

**Affiliations:** Texas State University, 601 University Dr, Encino 250, San Marcos, TX 78666 USA

**Keywords:** Electronic health records (EHR), Outcomes, Population health, Public health

## Abstract

Electronic health records (EHRs) have emerged among health information technology as “meaningful use” to improve the quality and efficiency of healthcare, and health disparities in population health. In other instances, they have also shown lack of interoperability, functionality and many medical errors. With proper implementation and training, are electronic health records a viable source in managing population health? The primary objective of this systematic review is to assess the relationship of electronic health records’ use on population health through the identification and analysis of facilitators and barriers to its adoption for this purpose. Authors searched Cumulative Index of Nursing and Allied Health Literature (CINAHL) and MEDLINE (PubMed), 10/02/2012–10/02/2017, core clinical/academic journals, MEDLINE full text, English only, human species and evaluated the articles that were germane to our research objective. Each article was analyzed by multiple reviewers. Group members recognized common facilitators and barriers associated with EHRs effect on population health. A final list of articles was selected by the group after three consensus meetings (*n* = 55). Among a total of 26 factors identified, 63% (147/232) of those were facilitators and 37% (85/232) barriers. About 70% of the facilitators consisted of *productivity/efficiency* in EHRs occurring 33 times, increased *quality* and *data management* each occurring 19 times, *surveillance* occurring 17 times, and *preventative care* occurring 15 times. About 70% of the barriers consisted of *missing data* occurring 24 times, *no standards* (interoperability) occurring 13 times, *productivity loss* occurring 12 times, and *technology too complex* occurring 10 times. The analysis identified more facilitators than barriers to the use of the EHR to support public health. Wider adoption of the EHR and more comprehensive standards for interoperability will only enhance the ability for the EHR to support this important area of surveillance and disease prevention. This review identifies more facilitators than barriers to using the EHR to support public health, which implies a certain level of usability and acceptance to use the EHR in this manner. The public-health industry should combine their efforts with the interoperability projects to make the EHR both fully adopted and fully interoperable. This will greatly increase the availability, accuracy, and comprehensiveness of data across the country, which will enhance benchmarking and disease surveillance/prevention capabilities.

## Introduction

### Background

Healthcare Information Technology (HIT) is changing how the healthcare industry operates and has already began to reduce waste and help improve health outcomes [1]. A major component of HIT is the Electronic Health Record (EHR). We used the definition of the EHR from the Center of Medicaid and Medicare Services (CMS): Electronic health records are digital forms of patient records that include patient information such as personal contact information, patient’s medical history, allergies, test results, and treatment plan [2]. Some benefits of EHRs include improving efficiency, increasing positive patient outcomes, and population health.^1^ Potential improvements in population health include EHRs ability to organize and analyze a large amount of patient information. This is particularly pertinent since the Public Health Data Standards Consortium (PHDSC) and the Center for Disease Control (CDC) completed its project to standardize public health case reports in accordance with HL7 [3]. This project in 2012 is one example of many ongoing efforts to establish data standards in support of the public health and the EHR.

Population health is “the health outcomes of a group of individuals, including the distribution of such outcomes within a group” [4]. and EHRs provide access to public health data to survey the population for potential health improvements or act as a safety net for potential health threats.^5^ A new program called “DiSTRIBuTE” that uses the EHRs in the surveillance of population health issues [5], and recent use found that electronic health records were better able to track “weekly influenza trends on an ongoing basis better than and in a “more timely than manual reporting from sentinel providers” [5]. Distributed Surveillance Taskforce for Realtime Influenza Burden Tracking and Evaluation (DiSTRIBuTE), run by the International Society for Disease Surveillance (ISDS), collects aggregated data by age group to improve decision making on public safety, cost, quality, and outcomes. This distributed-data is collected, analyzed, and interpreted in real time. Privacy of information is managed by the Fair Information Practice Principles (FIPPs), and the de-identified data is shared electronically to address specific population-health-related questions. The CDC in 2009 to support the tracking of the H1N1 pandemic, among other examples. EHRs can provide additional screening of health records beyond surveillance that can lead to additional research [5]. Public health surveillance observes a population and brings attention to various health threats or monitors the general health of the population [6]. There is even a positive correlation between the use of EHRs by primary care providers and the ability to accurately report to public health officials [7].

Utilizing and incorporating Electronic Health Records in surveillance and care interventions can help aid the health of the population it serves. Many of these studies have shown significant positive effects of EHRs interaction with public health. Previous research shows how EHRs are being used to surveil various populations, and some review other countries’ use of EHRs for surveillance [8]. Some positive effects that were observed included better surveillance of infectious diseases, improved management of patients with chronic diseases, and identify populations with higher risk factors [8]. The recent shifts in healthcare policy such as The ACA have recommended health practices to focus on preventive care to improve the overall health of the population [1]. Shih and De Leon discovered that physicians who implemented EHRs were better able to deliver recommended preventive care into their practices for low-income populations [9]. Electronic health records have been implemented to provide more coordinated and patient-centered care. EHR implementation in the ICU significantly reduces the central line associated bloodstream infections and surgical intensive care unit mortality rates [10]. EHRs provide secure access to patient information resulting in positive outcomes in relations to quality of care and productivity [11]. EHR systems have been used to manage chronic disease like diabetes, and it has been found that regular use of the EHR can reduce fragmentation of data and increase continuity of care between providers if the providers participate in health information exchanges [12]. EHRs in the emergency department (ED) improve medical decision making when using a decision tree; It increases the patient’s quality of life, and it was found to be cost-effective [13]. Another cost benefit assessment for using electronic health records for data showed promising results [14]. The European Electronic Health Records for Clinical Research (EHR4CR) has developed an innovative platform that is capable of transforming traditional research processes appeared to be highly beneficial by reducing the actual person-time, operational costs, or average cycle time for Phase II-III clinical trials when compared to current practices in a pre-launch environment [14].

Other studies have illuminated possible barriers to the success of EHRs. Some of these barriers include lack of interoperability, errors in medical information, and the financial resources that are required to accommodate HIT. Medical errors may still occur despite the increase of information being gathered from patients with the use of EHR [15]. Patients who received medical and surgical care showed same outcomes in six diverse states independent of the use of EHRs. No specific benefits in patient outcomes were related to EHRs [16]. Patient satisfaction can be adversely affected by the EHR due to a decrease in attention that a physician exhibits while making notes in the system [17]. Adoption of the EHRs is not without obstacles; however, results of the research is mixed on whether a proper implementation of an EHR could improve the operations of population health.

### Objectives

The purpose of this study is to review the literature previously published on the effects of EHRs on population health. Health Information Technology is becoming more widely utilized, however, the industry has still not been able to achieve its overall accessibility. It is our goal to answer whether the use of electronic health information can play a vital role in improving the health of populations, as well as identify key inhibitors to its adoption and/or key use.

## Methods

**Fig. 1:**
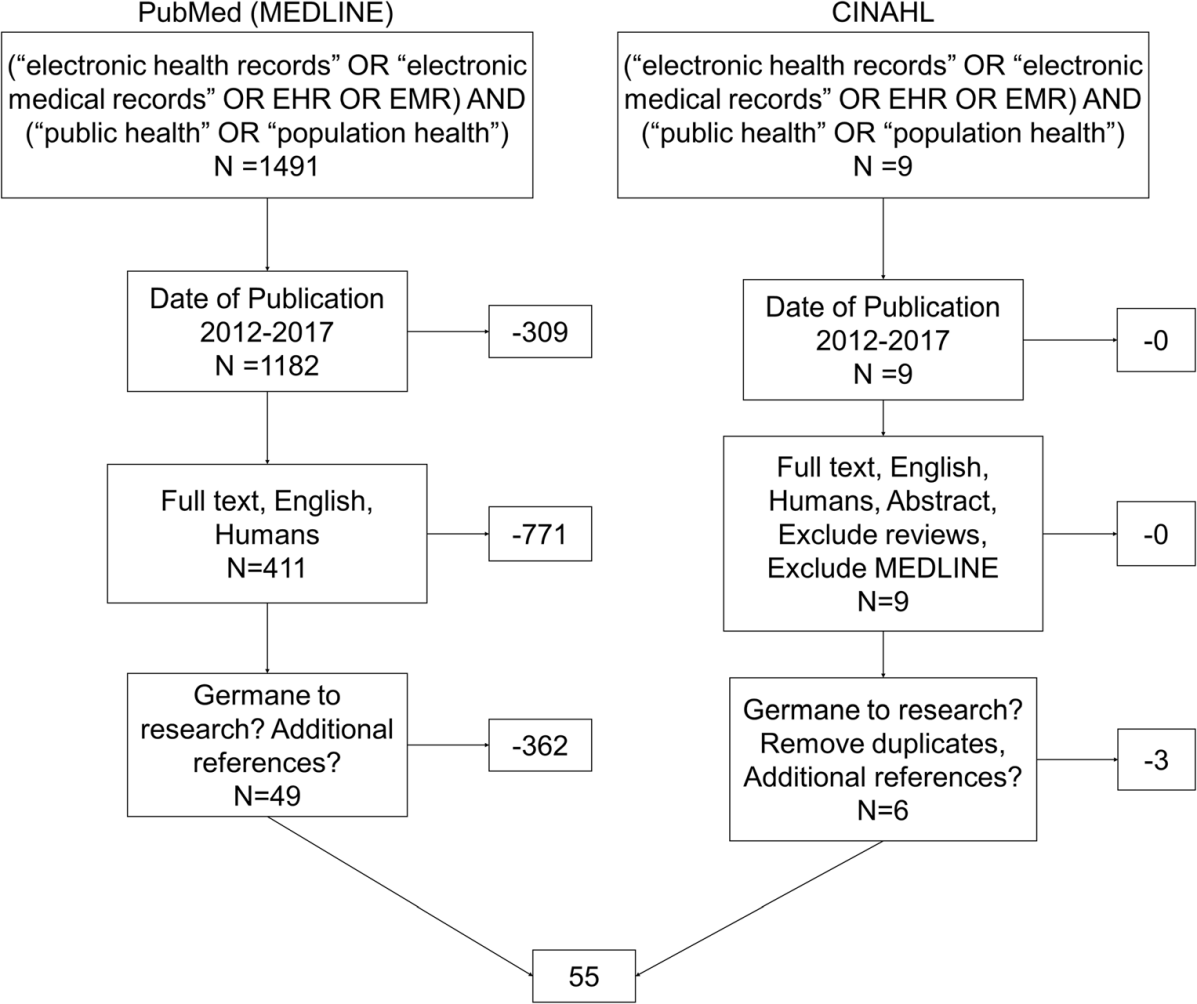
Literature Search with inclusion and exclusion criteria

The articles used for this systematic review were gathered and compiled using PubMed (MEDLINE complete) and The Cumulative Index to Nursing and Allied Health Literature (CINAHL). The search process is illustrated in Fig. 1. The United States National Library of Medicine’s Medical Subject Headings (MeSH) was used to find the key terms related to our topic in PubMed. With the help of MeSH, we were able to identify the appropriate sub-headings under the key terms. Our final key terms in the search process for both databases were “EHR” “electronic health record” “EMR” “electronic medical record” and “population health” or “public health”. While these terms have distinct definitions from each other, they are often used synonymously. We included both so that the search would be more exhaustive. In accordance with good research practice, we also included Boolean operators and quotation marks in the search string. The initial search in PubMed and CINAHL resulted in 1491 and nine items, respectively. We chose a timeframe of five years to keep the grouping small enough for reasonable analysis. After filtering relevant time frame academic journals, English only, and other peer review selection processes, we were left with 420 articles. Our process was to divide up these 420 abstracts between reviewers in a way that ensured each abstract was read by at least 2 reviewers. We independently assessed the relevance of each abstract in an Excel workbook and then combined the assessments during a consensus meeting. During this meeting we resolved any conflict in the assessments (germane or not germane to our research) to reach a final grouping of 55 articles for full analysis. A Kappa statistic of .83 was calculated, which demonstrates strong agreement among the reviewers, as well as consistency in reading and initial analysis of suitability. The same process was repeated for analysis of the articles that was used for analysis of the abstracts. Independent observations were recorded and later combined for a consensus meeting. During this second round, reviewers were also asked to pay attention to the references of each article to identify salient resources that may not have been caught by our search. This search did not result in any additional articles added to the group analyzed (*n* = 55).

During the second consensus meeting, reviewers shared their observations of facilitators and barriers to adoption of the EHR for managing public health. Through this process, reviewers categorized and grouped their observations in logical manner. An additional read of the articles took place to identify bias and limitations. These were shared in a third and final consensus meeting.

## Results

The results of our analysis are listed in Table 1. This table includes the source article, the facilitators, barriers, bias, and limitations of the articles analyzed.

**Table 1:** Summary of articles analyzed

**Author**	**Facilitator / enabler for adoption**	**Themes**	**Barrier to adoption**	**Themes**	**Bias or limitation**
Bailey, et al. [18]	- Increased utilization of prevention / primary care	Preventative Care	- None identified	None identified	- Limited sample from Oregon which means the results are not generalizable
	- Disease prevention	Preventative Care			
	- Utilization can be captured through EHR, even during dramatic upturns	Data Management			
	- Improved data quality	Quality			
	- Improved workflow	Productivity/ Efficiency			
	- Disease surveillance	Surveillance			
	- Improved timeliness	Quality			
Houser, et al. [19]	- Interoperability	Interoperability	- Lack of funding	Cost	- Limited sample from Alabama conference
	- Surveillance across all registries and all states	Surveillance	- Lack of medical staff support	Limited staff support	- Response bias
	- Advancing epidemiologic research	Data Management	- Changing data standards	No standards	- Lack of resources
	- Quality reporting	Quality	- Lack of full-time commitments	Critical thinking/treatment decisions	
	- Clinical decision support	Decision support	- Lack of standardized data exchange	No standards	
Metroka, et al. [20]	- Improved efficiency	Producivity/ Efficiency	- Records may be missing data	Missing data	- Limited external validity: This study was restricted to immunizations
	- Ease of use	Ease of use			
	- Data sharing	Data Management			
Blecker, et al. [21]	- Improved quality	Quality	- Data contains errors		- Limited external validity: Data only collected at one institution.
	- Ease of data collection	Data Management		Missing data / data error	
	- Ability to measure intensity of care	Productivity/ Efficiency			
Flood, et al. [22]	- Surveillance	Surveillance	- Missing data	Missing data / data error	- Measurement error can be mitigated with training.
	- Disease prevention	Preventative Care	- Human error in measurement	Missing data / data error	- Interrater reliability between systems needs to be measured and controlled.
					- EHR samples are convenience samples which may not be representative of the population.
Martelle, et al. [23]	- Improved accessibility	Productivity/ Efficiency	- Few incentives	Cost	- Small sample leads to low statistical power which reduces the external validity.
	- Improved quality of care	Quality	- Few inmates have email which reduces the demand for a patient portal.	Technology complex	- External validity limited: Study conducted in the correctional setting.
	- Financial assistance	Financial Assistance			
	- Interoperability	Interoperability			
Chambers, et al. [24]	- Improvement to quality	Quality	- None identified	None identified	- Selection bias
	- Surveillance	Surveillance			- Gender bias
	- Access to primary care information provides tailored quality improvement initiatives	Productivity/ Efficiency			- External validity limited because the gender/race demographics of the sample are not representative of the U.S.
Moody-Thomas, et al. [25]	- Improved primary care	Quality	- No independent method for determining the quality of data	No standards	- Quasi-experimental
	- Intervention effective in lowering the prevalence of tobacco	Health Outcomes			- Patient-reported behavior on bad behavior can be tempered to avoid uncomfortable discussions.
	- Disease prevention	Preventative Care			- No similar group exists for comparison of results.
	- Surveillance	Surveillance			
Vogel, et al. [26]	- Sustainability and generalizability	Quality	- Human error	Human error	- The voluntary nature of the Massachusetts League of Community Health Centers can create a fluid status of participating offices, which can also create orphaned data for queries.
	- Health outcomes	Health Outcomes	- Data is typically missing or incomplete	Missing data / data error	
	- Data management	Data Management	- Data error	Missing data / data error	
	- Productivity	Productivity/ Efficiency			
Calman, et al. [7]	- Improve surveillance and management of chronic disease	Surveillance	- Cost	Cost	- Not all primary-care entities cooperate and share with public health entities.
	- Efficiency	Productivity/ Efficiency	- No central agency mandating cooperation of public health with primary care entities.	No standards	
	- Interoperability	Interoperability			
	- Decisions about treatment	Decision support			
	- Disease prevention	Preventative care			
Duan, et al. [27]	- None identified	None identified	- Electronic system failures	Productivity loss	- Information bias caused by misclassification of errors.
			- Inaccurate data (data errors)	Missing data / data error	
			- Complexity	Technology complex	
Kawamoto, et al. [28]	- Disease prevention	Preventative care	- None identified		- Quasi experimental
	- Improved productivity	Productivity/ efficiency		None identified	- Control group comparison data were created using a model.
	- Improved efficiency	Productivity/ efficiency			
Behrens, et al. [29]	- Surveillance	Surveillance	- None identified	None identified	- Binning, as is common in Monte Carlo simulations, can cause bias in data.
					- Machine logic was used for best fit.
Cross, et al. [30]	- Support care coordination	Communication	- Interoperability	No standards	- Sample restricted to the state of Michigan.
	- Increased productivity	Productivity/ efficiency	- Cost	Cost	
	- Data management	Data management	- Total adoption is a barrier because some physicians don’t want to adopt unless referrals will have the technology.	Resistance to change	
	- Technology is up to date	Current technology	- EHRs can often obscure relevant information.	Missing data / data error	
Tanner, et al. [31]	- Patient safety for medications	Quality	- Fear of unintended consequences from EHRs.	Missing data / data error	- Selection bias: Only pre-meaningful use era adopters were queried.
	- Interoperability	Interoperability			- Does not address causation
	- Improved productivity	Productivity/ efficiency			
Emani, et al. [32]	- Decrease medical errors	Quality	- Resistance to change	Resistance to change	- Study was limited to two academic medical centers in one region.
	- Physician satisfaction	Satisfaction			- Did not include factors such as practice size.
	- Self-efficiency	Productivity/ efficiency			
Benkert, et al. [33]	- Overall positive impact overtime	Satisfaction	- Data failures/ challenges	Productivity loss	- Factors beyond the EHR that can affect poor outcomes were not measured.
	- Improved productivity	Productivity/ efficiency			- Data quality bias with level of user experience with the EHR.
	- Improved data collection	Data management			- Neither time lags nor staggered time points were measured or controlled.
Merrill, et al. [34]	- Improved efficiency	Productivity/ efficiency	- Structural limitation	Limited staff support	- The registry database limited comparison of EHR-submitted vs non-EHR submitted data.
	- Improved productivity	Productivity/ efficiency	- Missing data	Missing data / data error	
	- Improved compliance	Decision support			
	- Disease prevention	Preventative care			
Glicksberg, et al. [35]	- Disease prevention	Preventative care	- None identified	None identified	- External validity: One study group was not representative of the population.
McAlearney, et al. [36]	- Consistent communication	Communication	- Productivity loss during implementation	Productivity loss	- Small sample size greatly reduces statistical power and external validy.
	- Careful planning	Productivity/ efficiency	- Resistance to change	Resistance to change	
			- System failure	Productivity loss	
			- Poor computer skills	Limited staff support	
			- Slow queries	Productivity loss	
Polling, et al. [37]	- Data collection	Data management	- Missing data	Missing data / data error	- Not all data in the set could be matched with a record due to anonymity requirements. This limited the ability to compare data between records, and therefore limited the number of data points that were analyzed. These data points could have been dramatically different than those in the comparison.
	- Disease prevention	Preventative care			
Zhao, et al. [38]	- Data collection	Data management	- Interoperability	No standards	- Limited validity and reliability
Roth, et al. [39]	- Data collection	Data management	- Interoperability	No standards	- Data error was controlled by removing records that contained implausible values. This may have skewed the data because, while implausible, the data could have described an unusually sick population.
	- Surveillance	Surveillance	- Prone to data-entry error	Missing data / data error	- Free-text fields are inherently difficult to include in analysis. The data contained within free-text fields may have skewed the results differently.
			- Missing data	Missing data / data error	- Without a time-series or longitudinal study, it is difficult to generalize the results.
Barnett, et al. [40]	- None identified	None identified	- none identified	None identified	- The small sample of 17 hospitals reduces statistical power which may limit the generalizability of the results.
					- Researchers unable to explore the associataion of EHR implementation with inpatient outcomes stratified by implementation context, hospital, or EHR characteristics.
Drawz, et al. [41]	- Improved performance	Productivity/ efficiency	- Limited functionality	Technology complex	- The lack of nationwide data eliminates comparisons to a national benchmark.
	- Interoperability	Interoperability			
	- Improve measuring data (data collection)	Data management			
Thirukumaran, et al. [42]	- None identified	None identified	- Temporary decrease in quality	Productivity loss	- Limited generalizability
Adler-Milstein J, Everson J, Lee SY. [43]	- Increased quality	Quality	- None identified	None identified	- Adherence to process measurers was high across hospitals which reduces the opportunity to observe EHR-driven improvements.
	- Increased efficiency for hospital care	Productivity/ efficiency			- This study only analyzed the Medicare arm of the Meaningful Use program.
	- Patient satisfaction	Satisfaction			
	- positive relationship between EHR adoption and performance	Interoperability			
Ananthakrishnan, et al. [44]	- Health outcomes	Health outcomes	- Misclassification (data error)	Missing data / data error	- The cohort studied represents a small population: Therefore, the external validity of results are limited.
	- Quality in documentation	Quality	- Interoperability	No standards	- All provider notes may not have been captured if a participant saw a physician through a private setting.
	- Lends generalizability to findings	Productivity/ efficiency			
Carayon, et al. [45]	- Increased productivity	Productivity/ efficiency	- Increased amount of time spent on documentation and clinical review	Productivity loss	- Not generalizable nationwide because data were collected at only one location.
	- Efficiency gains	Productivity/ efficiency	- Decreased direct patient care (quality)	Decreased quality	- Physicians were not identified, and therefore their contributions may have occurred both pre and post treatment. Having this information would have made analysis easier. This can introduce observer bias that has not been controlled for.
Redd, et al.	- None identified	None identified	- Negative impact on productivity and efficiency	Productivity loss	- Face validity: Clinical volume is not an exact match for provider productivity, but other studies have used this measure.
[46]			- Time consuming	Technology complex	- Construct validity: Due to the lack of baseline data available, it is difficult to discern that the intended measure is accurate.
			- Missing data	Missing data / data error	
Jones & Wittie [47]	- Widespread adoption	Current technology	- Lacked functionality	Accessibility/ utilization	- Limited external validity due to the uncertainty that Beacon communities across the country are homogeneous.
	- Improved quality	Quality	- Complexity	Technology complex	- Self-report data can be questionable, but sufficient research has been conducted using similar data, researchers felt comfortable.
	- Care coordination (communication with data exchange)	Interoperability			
	- Layering of financial incentives	Financial assistance			
	- Technical assistance	Communication			
Hammermeister, et al. [48]	- Data collection	Data management	- Missing data	Missing data / data error	- Limited clinic-level data precludes comparison characteristics between hih and low outlier clinics.
	- Inexpensive data collection (cost)	Financial assistance			- External validity is limited because the sample is not representative of the national population.
Benson, et al. [49]	- Interoperability between EHR and primary care systems	Interoperability	- Potential missing data	Missing data / data error	- Infrequency of visits creates missing data.
	- Efficient comparison of patients	Productivity/ efficiency	- Some privacy concerns	Privacy concerns	- Standardized measures for risk factors do not exist.
			- Inability to conduct certain logistic functions (lack of functionality)	Productivity loss	- Self-report data can be unreliable.
Soulakis, et al. [50]	- Communication between patients and providers	Communication	- Complex analysis	Technology complex	- The measure of interrater reliability is confounded because some providers served on many teams.
	- Preventative care	Preventative care			
Burke, et al. [51]	- Improved over quality of outpatient clinical notes	Quality	- Standards across EHRs		- Not generalizable to all EHR systems because only one was studied.
	- Accessibility	Ease of use		No standards	
	- Improved efficiency	Productivity/ efficiency			
Keck, et al. [52]	- Surveillance	Surveillance	- Limited design, deployment and function (complexity)		- Construct validity is questionable due to lack of baseline data.
	- Increased time availability (productivity)	Productivity/ efficiency		Technology complex	- Generalizability limited because only the Indian Health System was studied.
	- Improved data validity and reliability	Quality			
Roth, et al. [53]	- Surveillance	Surveillance	- Fail to capture important discrete necessary data (missing data)	Missing data / data error	- Selection bias due to a convenience sample.
	- Reduce health disparities (health outcomes)	Health outcomes	- Lack of workflow integration paradigms(productivity)	Productivity loss	- Smoking data is inherently underreported, so the effects of this study are understated.
De Moor, et al. [54]	- Reduce duplication and errors	Quality	- Regional diversity in languages.	No standards	- None identified
	- Data collection	Data management	- Interoperability	No standards	
	- Improved efficiency	Productivity/ efficiency	- Inconsistent documentation	Missing data / data error	
			- Data quality	Decreased quality	
Chang, et al. [55]	- None identified	None identified	- Missing data	Missing data / data error	- External validity limited: While the computed algorithm satisfactorily predicted one behavior, it is uncertain if such models can be developed for all.
Reed, et al. [56]	- Increased/positive impact on critical thinking skills	Decision support	- None identified	None identified	- Response bias decreased the number of participants.
Inokuchi, et al. [57]	- Productivity (reduced time)	Productivity/ efficiency	- No patient outcomes		- Need larger sample size
	- Increased physician satisfaction	Satisfaction		Decreased quality	- The Hawthorne effect may have increased bias toward the new EMR.
	- Increased use of information	Decision support			- External validity may be questionable because only one EMR was studied.
	- Organizational impact	Productivity/ efficiency			
Silfen, et al. [58]	- Prompt healthcare providers to screen for chronic health issues (preventative care)	Productivity/ efficiency	- No return on investment		- External validity limited because data were not available for all organizations and anything outside of New York City.
	- Facilitate provider referrals	Communication		Cost	- Data were not complete
	- Supplies rapid feedback to providers	Decision support			
	- Track patient outcomes	Health outcomes			
	- Monetary/financial incentive	Financial assistance			
Zera, et al. [59]	- None identified		- No effect on the rates of diabetes screening	Disease management	- Data bias may have skewed results toward the null result.
		None identified	- No access to screening responses (structural limitation)	Missing data / data error	- External validity limited: Small numbers in the control group reduces the statistical power.
			- No patient outcomes	Decreased quality	
Baus, et al. [60]	- Surveillance	Surveillance	- The EHR is designed for patient care, not for research.	Accessibility/ utilization	- Not generalizable, sample bias: Clinics were chosen through purposive sampling.
	- Preventative care	Preventative care	- Human error in recording data in the EHR.	Human error	- Unable to combine data for extrinsic information (structural limitation).
	- Data quality for population health management	Quality			
Baus, et al. [61]	- Preventative care	Preventative care	- Difficulty of extracting necessary data (technical challenges).	Technology complex	- Limited variability in participants limits external validity.
	- Surveillance	Surveillance	- Cost	Cost	
	- Improved efficiency	Productivity/ efficiency	- Interroperability	No standards	
	- Improve decision support	Decision support			
	- Increase the application of patient data to care	Data management			
	- Improve health outcomes	Health outcomes			
Haskew, et al. [62]	- Real time access (efficiency)	Productivity/ efficiency	- Cost	Cost	- Limited external validity due to short time studied and difficulty of implementation model.
	- Sharing data (communication)	Communication	- Limited staff	Limited staff support	
Puttkammer, et al., [63]	- Preventative care	Preventative care	- Missing data		- Self-report data is questionable and subject to ability to recall or social desirability.
	- Data/information accessibility	Ease of use		Missing data / data error	- Missing data that could have skewed the results.
					- External validity limited because only two organizations studied.
Zheng, et al. **66]**	- Surveillance	Surveillance	- Difficulty combining information from EHR with structured data	Technology complex	- External validity limited
	- Data collection	Data management			
Wu, et al. [64]	- Data collection	Data management	- Missing data on smoking status		- Citation bias
	- Smoking surveillance	Surveillance		Missing data / data error	- Self-report data on smoking is limited due to social desirability, therefore the results of this study may be understated.
	- Facilitating care identification	Communication			
Nguyen & Yehia [65]	- Data collection	Data management	- Different documentation rates at Different healthcare systems	No standards	- External validity limited because only one health system in one region of the U.S. was studied.
	- Preventative care	Preventative care			
Tomayko, et al. [66]	- Data collection	Data management	- None identified	None identified	- External validity limited because the demographics do not match that of the U.S.
	- Preventative care	Preventative care			- Self-report data can be questionable and subject to bias due to recall and social desirability.
	- Disease management/monitoring (child obesity)	Surveillance			
	- Quality improvement	Quality			
	- Greater surveillance of a population	Surveillance			
	- Cost effective	Financial assistance			
Romo, et al. [67]	- Surveillance	Surveillance	- Data is often skewed toward those who seek care.	Missing data / data error	- Data bias: Missing values were filled with estimates which may skew the results.
	- Generalizability	Productivity/ efficiency			- Self-report data is questionable and subject to bias due to recall and social desirability.
					- External validity limited to U.S. only.
Chambers, et al. [68]	- Data collection	Data management	- None identified	None identified	- Quasi experimental.
					- Selection bias.
Wang, et al. [69]	- Improved quality	Quality	- None identified	None identified	- External validity limited: Only 151 organizations studied, therefore generalizing outside those practices may be limited.
	- Work flow variability (productivity)	Productivity/ efficiency			- Structural limitation
					- Selection bias: Early adopters were selected for the study.
Chiang, et al. [70]	- Increased faculty providers	Satisfaction	- Initial decrease in clinical volume	Productivity loss	- Interrater reliability was controlled by using a stable group of providers.
	- Longer notes	Communication	- Increased time expenditure and documentation times	Technology complex	- Baseline data was established during a three-month period (Nov-Jan).
	- More automatically generated texts (efficiency)	Productivity/ efficiency	- Increased reliance on textual descriptions and interpretations (human error)	Human error	- Construct validity limited because clinical volume may not be an equal measure of productivity.
			- Little to no increase in clinical volume	Productivity loss	- External validity limited: The only EHR studied was at a large academic medical center which may not be representative of all organizations in the U.S.

We examined the work of 414 authors and co-authors who published 55 works that discuss Electronic Health Records, Population and or Public Health. We identified a total of 232 factors, which consisted of 63% (147/232) facilitators and 37% (85/232) barriers. Utilizing EHRs resulted in a greater number of benefits than negative impacts to population health. During the review process, various aspects of electronic health records showed that the utilization of these HIT improves population and public health. Benefits of using electronic medical records describe how EHRs improved the productivity and efficiency of health organizations to better serve populations. Increased healthcare access to individuals provides more comprehensive documentation from the population from the surveillance of public health screening and preventative care. Electronic health records allow health professionals to share and incorporate more public health information among various providers. This improves the population’s ability to survey the populations for chronic disease, contagious infections, and allows for more rapid and uniform transference of patient information [7, 18–71]. The incorporation of new technology is expected to have some flaws associated with its integration into the healthcare field [7, 18–71]. Some of the major setbacks of EHRs and EMRs include a temporary decrease in productivity, while staff and medial personal incorporate and train employees to use an entirely new system. Alongside with new operational systems medical efforts, lack of functionality, system failures, and simple resistance to change by providers can occur. These can have negative impacts on public health as missing or incorrect information can be transmitted for surveillance. Other barriers include the inability to generalize one healthcare organization’s experience to others due to various types of EHRs and systems to the wide variety of populations and settings. Some healthcare populations have been found to be more accepting of EHRs while others have found it more difficult to incorporate them into a daily routine [1]. The authors were able to organize and examine these themes in the discussion section.

### Additional analysis

Affinity matrices were created to further analyze facilitators and barriers. These matrices are illustrated in Table 2.

**Table 2:** Affinity matrix of facilitators and barriers

Facilitators	Reference	Occurrences		Reference	Barriers
Productivity/ efficiency	19,21,22,24,25,27,28,30*,32,33–36*,38,43,45–47*,51,53-56,59*,60, 63,64,70,72,73	33	24	21,22,23*,27*,29,32,33,36,39,41*,46,48,50,51,55,56,57,61,65,67,70	Missing data / data error
Quality	19*,20,22,24-27,33,34,45,46,49, 53,54,56,62,69,72	19	13	20*,26,28,32,40,41,46,53,56*,63,68	No standards
Data management	19–22,27,28,32,35,39, 40,41,43,50,56,66-69,71	19	12	29,35,38*,44,47,48,51,55,73*	Productivity loss
Surveillance	19,20,23,25,26,28,31,41,54,55, 62,63,66,67,69*,70,	17	10	24,29,43,48,49,52,54,63,66,73	Technology complex
Preventative care	19*,23,26,28,30,36,37,39,52,62, 63,65,68,69	15	7	20,24,28,31,60,63,64	Cost
Communication	32,38,49,52,60,64,67,73	8	4	47,56,59,61	Decreased quality
Interoperability	20,24,28,33,43,45,49,51	8	4	20,36,38,64	Limited staff support
Decision support	20,28,36,58-60,63	7	3	32,34,38	Resistance to change
Health outcomes	26,27,46,55,60,63	6	3	27,62,73	Human error
Satisfaction	34,35,45,59,73	5	2	49,62	Accessibility/ utilization
Financial assistance	24,49,50,60,69	5	1	61	Disease management
Ease of use	21,53,65	3	1	20	Critical thinking/treatment decisions
Current technology	32,49	2	1	51	Privacy concerns
* more than one occurrence		147	85		
None identified	29,42,44,48,57,61			19,25,30,31,37,42,45,58,69,71,72	

**Fig. 2:**
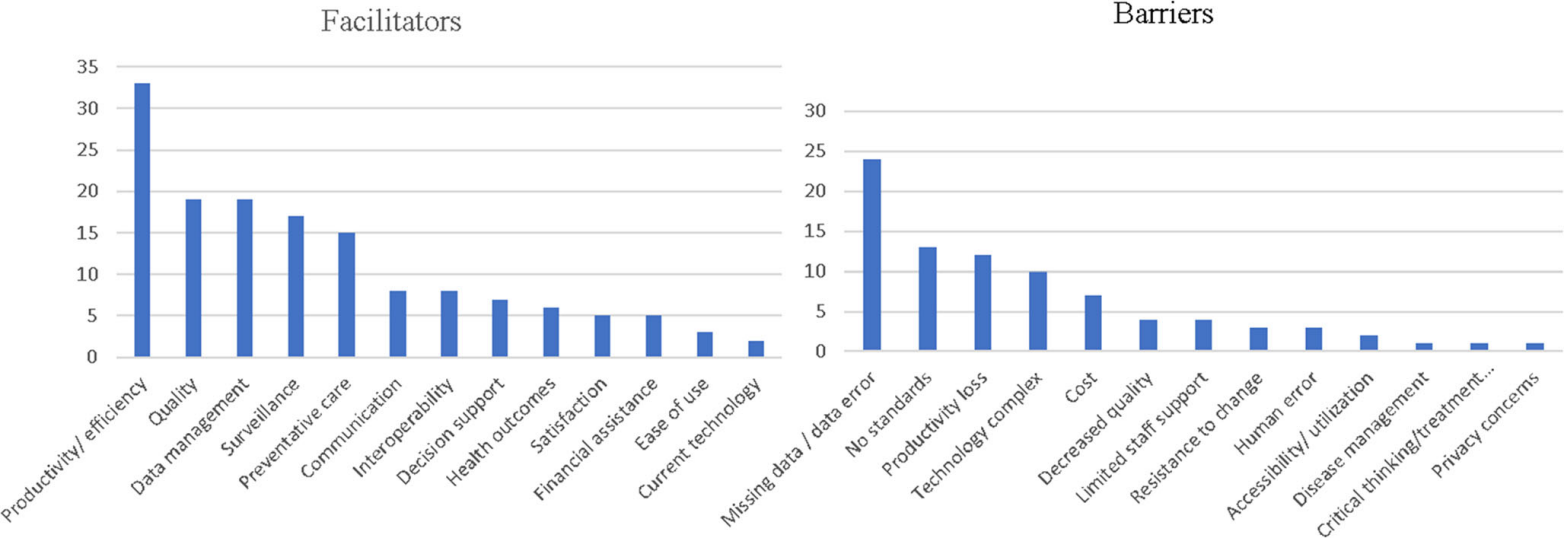
Charts of the frequency of facilitators and barriers

A visual representation of these factors can also be seen in fig. 2.

Thirteen facilitators and 13 barriers were identified. The occurrences of facilitators outweighed those of the barriers 3:2. Several facilitators and barriers were similar and were combined, for instance productivity and efficiency. Articles that mentioned both facilitators are marked in the tables with an asterisk. Facilitators identified are *productivity/efficiency* [19,21,22,24,25,27,28,30*,32,33–36*,38,43,45–47*,51,53-56,59*,60,63,64,70,72,73]*,quality* [19*,20,22,24-27,33,34,45,46,49,53,54,56,62,69,72]*,data management* [7, 18–21, 26, 30, 33, 37–39, 41, 48, 54, 64–66, 68, 71]*, surveillance* [19,20,23,25,26,28,31,41,54,55,62,63,66,67,69*,70]*, preventative care* [19*,23,26,28,30,36,37,39,52,62,63,65,68,69]*, communication* [30, 36, 47, 50, 58, 62, 64, 70]*, interoperability* [7, 19, 23, 31, 41, 43, 47, 49]*, decision support* [7, 19, 34, 56–58, 61]*, health outcomes* [25, 26, 44, 53, 58, 61]*, satisfaction* [32, 33, 43, 57, 70]*, financial assistance* [23, 47, 48, 58, 66]*, ease of use* [20, 51, 63]*, and current technology* [30, 47]*,* Barriers identified are *missing data/data error* [21–23*,27*,29,32,33,36,39,41*,46,48,50,51,55-57,61,65,67,70]*, no standards* (of data or interoperability) [20*,26,28,32,40,41,46,53,56*,63,68], *productivity loss* [29,35,38*,44,47,48,51,55,73*]*, technology (too) complex* [23, 27, 41, 46, 47, 50, 52, 61, 70, 71]*, cost* [7, 19, 23, 29, 58, 61, 62]*,decreased quality (of data or care)* [45, 54, 57, 59]*, limited staff support* [19, 34, 36, 62]*, resistance to change* [30, 32, 36]*, human error* [26, 60, 70]*, accessibility/utilization* [47, 60]*, disease management* [59]*, critical thinking/treatment decisions* [19]*, privacy concerns* [49]*.* The top five facilitators and top four barriers make up about 70% of the factors observed.

## Discussion

### Summary of evidence

In this systematic review the authors reviewed 55 articles. The analysis identified 13 facilitators and 13 barriers, and facilitators outweighed barriers 3:2. The top three facilitators were an increase in *productivity/efficiency* (greater capacity, more efficient procedures and processes, etc.), an increase in the *quality* of data or care (data that was more accurate, more precise, and contained less error; care that produced higher quality outcomes as a result of more accurate data), and various aspects of *data management* (users were able to access patient data in a more efficient manner). The top three barriers were *missing data* (some data was missing or was not filled in) */ data error* (incorrect data was entered), *no standards* for interoperability (data could not easily be shared between providers), and a *loss of productivity* (teaching users how to use the EHR and data-entry requirements were time consuming and took users away from other duties in the office, which made the office less productive). The results of this review show more positive than negative factors for the use of the EHR to manage public health and surveillance.

The facilitator most often found in the literature is the increase of either productivity, efficiency, or both. Organizations were maximized time with patients instead of writing documentation. These articles said that EHRs improved the workflow in organizations. Other organizations identified a loss in productivity for the same reason. This could have been due to the stage of implementation in which the organizations were.

With the ability to access a greater number of records in a more productive way, it was not surprising that surveillance accounted for the third most recorded facilitator. Surveillance can utilize information from EHRs to make population and public health predictions as well as track occurrences of infectious diseases and other public health functions to have a better overall review of a population’s health.

### Limitations

To control for selection bias, reviewers agreed on definitions and concepts prior to the search and analysis of articles. Each article was reviewed and analyzed by multiple reviewers. A series of consensus meetings was held to share observations and agree on next steps. The team calculated a *Kappa* statistic of 0.83 which in fact shows a high level of agreement.

Publication bias is likely to occur because publishers tend to publish articles with significant relationships, and therefore articles that did not result in significant findings were not able to be selected for this review [72]. Our search was limited to PubMed and CINAHL, which may have impacted the scope of our results. These databases were chosen for their comprehensive scope and positive reputation in research.

### Comparison to other research

Contrary to studies on the adoption of the EHR, the authors found that cost was not as prevalent a barrier in using EHRs in support of public health. This could be a result of sufficient time passing for financial incentives to alleviate the concern. The articles reviewed intimated that EHRs were cost effective, enhance productivity/efficiency and quality, and they are conducive for data collection when missing data is analyzed. Standards for interoperability need to continue to progress: Until all EHR solutions reach the same level of interoperability, data sharing cannot be assured.

## Conclusion

Additional research should follow from this review. Productivity was both a facilitator and a barrier. It would be interesting to know if the latter is during implementation and the former is after. As nationwide adoption of a fully interoperable EHR progresses, many barriers identified in this review such as standards, and resistance to change could be mitigated. As more data becomes available through the EHR, relationships to outcomes should appear. Appropriate training on EHRs use, may help with the level of complexity among health care providers and their staff.

The EHR can improve health care productivity and efficiency to better serve public health. An abundance of health care information can be managed through databases by using electronic medical records, and this makes data more easily shared between providers and organizations.
